# Impact of *Herpes simplex virus* load and red blood cells in cerebrospinal fluid upon herpes simplex meningo-encephalitis outcome

**DOI:** 10.1186/1471-2334-12-356

**Published:** 2012-12-18

**Authors:** Julien Poissy, Karen Champenois, Anny Dewilde, Hugues Melliez, Hugues Georges, Eric Senneville, Yazdan Yazdanpanah

**Affiliations:** 1 Service universitaire de Maladies infectieuses et du Voyageur, Centre hospitalier de Tourcoing, France; 2 Equipe ATIP/Avenir INSERM U995, Université Lille Nord de France, Lille, France; 3 Laboratoire de Virologie, CHRU de Lille, France; 4 Service de Réanimation polyvalente et des Maladies infectieuses, Centre hospitalier de Tourcoing, France

**Keywords:** Herpes virus, Prognosis, Neurological/brain, Viral load

## Abstract

**Background:**

Herpes simplex encephalitis (HSE) often leads to severe disability or death. Factors usually associated with outcome include Simplified Acute Physiology Score, age and delay of initiation of acyclovir treatment.

Our aim was to determine the impact of *Herpes simplex virus* (HSV) load in cerebrospinal fluid (CSF) upon HSE outcome.

**Methods:**

We retrospectively determined HSV load in the CSF of 43 patients with confirmed HSE, hospitalized in northern France from 1998 to 2005, using CSF samples collected the day of hospital admission and stored at −20°C. We analyzed the association between HSV load and mortality/morbidity by the Glasgow Outcome Scale. Fisher’s exact test and Wilcoxon’s test were used for statistical analysis.

**Results:**

The M/F sex ratio was 1.7 and median patient age was 61 years. Median HSV load in CSF was 2.0 log copies/μL (IQR 25-75=1.2-2.6). The mortality rate was 32.6% six months after HSE diagnosis. Higher age was associated with mortality (*p*=0.03). Longer delay in acyclovir initiation tended to be associated with higher mortality but did not reach statistical significance (*p*=0.08). Severe disability and death due to HSV were associated with a higher Knaus score (*p*=0.004), later acyclovir initiation (p=0.006), older age (*p*=0.04) and presence of red blood cells in CSF (p=0.05). HSV load in CSF was neither associated with mortality (*p*=1.00) nor with morbidity (*p*=0.90).

**Conclusion:**

In this study, HSV load in CSF was not found to be associated with poor outcome in patients with HSE. These data do not support measurement of HSV load at admission in patients with HSE.

## Background

*Herpes simplex virus* (HSV) is the primary cause of viral necrotizing encephalitis in developed countries; when untreated, the disease has a 70% mortality rate [1,2]. Despite the introduction of acyclovir treatment, mortality and persistent neurologic impairment due to HSV encephalitis (HSE) remain high [3,4]: 15 to 20% of patients with HSE die and about 60% of surviving patients develop long-term neurological sequels [1,5]. Therefore, it is important to identify factors associated with severe morbidity and mortality in HSE.

Factors commonly reported to be associated with HSE outcome are age, level of consciousness at initial management of the patient [6] and time to start of acyclovir treatment [5]. Previous histopathological descriptions of HSE also showed a relationship between a high number of viral particles upon brain biopsy and poor disease outcome [7]. However, since HSV load in the cerebrospinal fluid (CSF) is not routinely determined, data on the impact of high HSV load upon HSE outcome are scarce and contradictory [8-11].

The aim of this study was to assess the association between HSV load in CSF (before initiation of acyclovir treatment) and HSE outcome.

## Methods

### Patients

We conducted a retrospective study in patients diagnosed with HSE in northern France from 1998 to 2005. We focused on patients for whom a CSF sample was available prior to acyclovir treatment initiation in order to assess the association between CSF HSV load and morbidity and mortality six months after HSE onset.

### Data collection

Data were collected retrospectively from patient medical records using a standardized questionnaire. Morbidity was assessed using the Glasgow Outcome Scale (GOS) divided into 5 categories (1: good recovery without neurologic impairment; 2: mild disability; 3: moderate disability; 4: severe disability; 5: death) [12,13]. The following informations were collected: i) at treatment initiation in the hospital: age, sex, alcohol consumption, severity of underlying disease, body temperature and Glasgow coma scale (GCS [13]); ii) during patient stay in the hospital: CSF cell counts prior to acyclovir initiation and time period between hospital admission, lumbar puncture and acyclovir initiation. Severity of the underlying disease was determined using the Knaus scale (A: normal health status; B: moderate limitation (impossible to work or study); C: severe limitation (need to have a third person in daily life); D: bedridden patient) and the MacCabe score (0: no fatal disease; 1: death in the next five years; 2: death in the following year) [14,15].

### Measurement of HSV load in CSF

HSV load was measured in CSF of patients with HSE using a frozen sample taken before acyclovir treatment was initiated. Samples were prospectively collected and stored at −20°C. When frozen CSF was available (n=30), we extracted HSV DNA using the QIAamp® DNA Blood Qiagen kit according to the manufacturer’s instructions. We performed the extraction and quantified HSV load on 200 μL of the sample for 6 patients and less than 200 μL for 24 patients. When no CSF was available to extract DNA (n=13), we quantified the viral load directly on DNA that had been extracted on the date of sampling and stored frozen at −20°C. Then, quantification of the viral load was performed using the artus® HSV-1/2 LC PCR kit manufactured by Qiagen. This PCR kit uses real time PCR for quantification and contains a second system of heterologous amplification to monitor for the possible presence of inhibitors. It allows the distinction between HSV1 and 2.

### Statistical analysis

First, we used descriptive statistics to evaluate patient characteristics according to CSF sample availability. Secondly, we assessed univariate associations between HSV load (or other variables collected) and HSE outcome defined as mortality at six months and morbidity at six months (low morbidity: GOS 1, 2 and 3 versus high morbidity: GOS 4 and 5).

Non-parametric tests were used for all comparisons. Fisher’s exact test and Wilcoxon’s test were used to compare qualitative and quantitative variables, respectively. A significance threshold of 0.05 was retained. Statistical analysis was performed using SAS software (SAS 9.1, SAS Institute Inc., Cary, NC, USA).

### Ethics statement

All patients included in the study were followed in different hospitals from northern France. The care practices included CSF sampling for diagnosis and management of these patients with suspected HSE. HSV qualitative PCR were performed routinely for this diagnosis. We performed quantitative PCR retrospectively on the residual frozen CSF. No additional sampling was necessary. This study was approved by the “Comité de Protection des Personnes Nord Ouest IV”, the ethic committee of Lille Universitary Hospital.

## Results and discussion

Between 1998 and 2005, 78 patients sought care for HSE in northern France. Those patients belonged to a previously published cohort [16]. Frozen CSF samples were available for 43 of them. All of these were from patients with HSV-1 encephalitis. Characteristics of these patients are presented in Table 1. Mortality at 6 months was quite high (32.6%) and severe morbidity (GOS score 4 and 5) was frequent (44.7%). This was not significantly higher than mortality of non included patients (20.0%) (p=0.12). Included and not included patients did not differ for other demographic, clinical or biological main characteristics (data not shown). Characteristics usually linked to poor prognosis in the literature were found in our cohort., i.e. age, alcohol consumption, time period between admission and acyclovir introduction (Table 1, [1,2,5,6,17,18]).

**Table 1:** Characteristics of patients with CSF sample in our cohort

	**Patients with CSF sample (n=43)**	
***Demographic characteristics***		
Age in years (median, [IQR])	61	[50–69]
Sex (n, % of males)	27	62.8%
***Mortality***		
Death at 6 months (n, %)	14	32.6%
***Morbidity***		
Glasgow Outcome Scale, GOS (n, %)		
Good recovery to moderate disability (1–3)	21	55.3%
Severe disability and death (4–5)	17	44.7%
***Other characteristics***		
MacCabe score (n, %)		
No fatal disease (0)	34	79.1%
Death in the next 1 or 5 years (1–2)	8	19.0%
Knaus score (n, %)		
Normal health status (A)	23	54.8%
Moderate limitation to bedridden patients (B-D)	19	45.2%
Time to acyclovir initiation in days (median, [IQR])	1	[0–2]
Alcohol consumption (n, %)	6	14.0%
Body temperature (°C) (median, [IQR])	38.9	[38.4–39.5]
GCS score (median, [IQR])	14	[12–15]
Leukocyte count (cells/μL) (median, [IQR])	50	[8–142]
Lymphocyte percentage of white cells (median, [IQR])	80.5	[10–96]
Time to lumbar puncture (days) (median, [IQR])	1	[0–1]
HSV load (log10 copies/μL) (median, [IQR])	2.0	1.2–2.6

*IQR*: Interquartile range.

There are some missing data for GOS, Mac Cabe and Knaus scores and time to acyclovir initiation.

**Figure 1:**
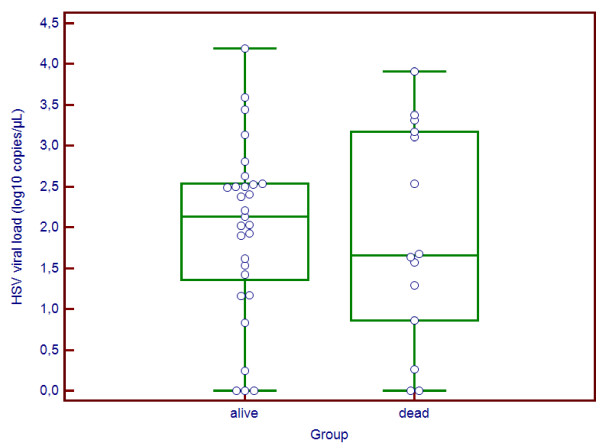
Distribution of CSF HSV viral loads by vital status in 43 patients with HSV encephalitis and an available frozen CSF sample.

We first studied factors associated with mortality six months after HSE onset (Table 2). Median CSF HSV load was 2.1 log10 copies/μL in patients who survived vs. 1.7 log10 copies/μl in patients who had died at six months (Figure 1). The difference was not significant (p=0.95) and we observed a great dispersion of the viral load values. Older age was significantly associated with mortality at six months (71 years in deceased patients vs. 59 years in patients who survived, p=0.03). There was a trend toward an association between mortality at six months and longer time between admission and start of acyclovir treatment (2 days in deceased patients vs. 0 day in patients who survived, p=0.08).

**Table 2:** Univariate analysis of factors associated with HSE mortality and morbidity

**Variables**	**Mortality (n=43)**			Morbidity (n=39*)		
	**Alive (n=29)**	**Dead (n=14)**	***p***	**Low (n=17) GOS 1-3**	**High (n=22) GOS 4-5**	***P***
Median HSV load (Log_10_ copies/μL)	2.1	1.7	0.95	2.0	1.8	0.95
Age in years (median)	59	71	0.03	57	64	0.04
Knaus score (%)			0.07			0.004
Normal health status (A)	64.3	35.7		81,3	18.8	
Moderate limitation in bedridden patients (B-D)	35.7	64.3		33.3	66.7	
Lymphocytic CSF (%)	66.7	41.7	0.17	80.0	42.1	0.04
Median blood red cell counts in CSF (cells/μL)	4	49	0.17	0	49	0.05
Median time to lumbar puncture (days)	0	1	0.23	0	1	0.02
Median time to acyclovir initiation (days)	0	2	0.08	0	2	0.006

* Morbidity analysis was performed for 38 patients, since Glasgow outcome scale was not available for five patients.

*GOS 1–3*: Glasgow outcome scale, classes 1 to 3: good recovery to moderate disability.

*GOS 4–5*: Glasgow outcome scale, classes 4 and 5: severe disability and death.

**Figure 2:**
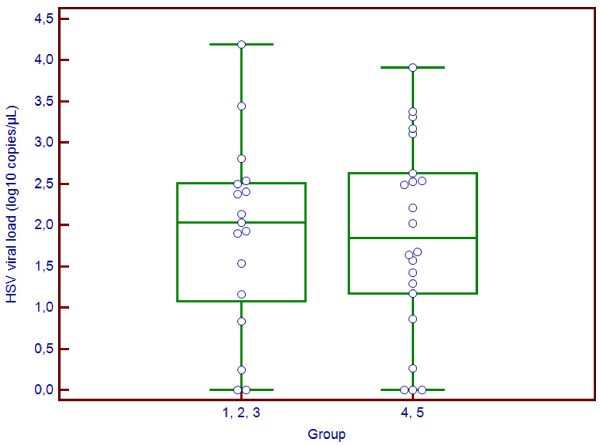
Distribution of CSF HSV viral loads by Glasgow Outcome Scale in the 39 patients for which data were available.

We next studied factors associated with morbidity six months after HSE onset (Table 2). Results were available for 39 patients, as the functional status was unknown for 4 patients. CSF HSV load distribution by GOS class is shown in Figure 2. Median HSV load in CSF was 2.0 log10 copies/μL in patients with low morbidity (GOS 1, 2 and 3) versus 1.8 log10 copies/μL in patients with high morbidity (GOS 4 and 5). The difference was not significant (p=0.95). Factors significantly associated with severe morbidity were: older age (64 vs. 57 in patients with low morbidity, p=0.04), delayed lumbar puncture (1 day vs. 0 day, p=0.02), delayed acyclovir introduction (2 vs. 0 days, p=0.006), a worse Knaus score (A: 18.8% vs. 81.3%; B+C+D: 66.7% vs. 33.3%, p=0.004) and the presence of red blood cells in the CSF sample (49 cells/μL vs. 0, p=0.05). A predominance of lymphocytes in the CSF was associated with lower morbidity (42% in patients with high morbidity vs. 80% in those with low morbidity, p=0.04).

We assessed factors associated with HSE outcome in 43 patients with symptomatic HSV encephalitis who sought care in northern France between 1998 and 2005. To our knowledge, this cohort is the largest ever used for evaluating the impact of HSV load in CSF upon HSV encephalitis outcome. Moreover, we used a technique of quantitative real time PCR, limiting the risks of false positive samples. We found no association between HSV load and HSE outcome, nor in mortality or morbidity six months after HSE onset.

Our results are consistent with other studies concerning factors usually associated with poor outcome in HSV encephalitis: older age, delayed initiation of acyclovir treatment and a high Knaus score [1,2,5,6,17,18].

Regarding the absence of association between CSF HSV load and HSE outcome, our results are in contrast to results reported by Dominges *et al.*[8]*.* However, in that study, HSV load was studied as a dichotomous variable with a cut-off of 100 copies/μL separating high and low viral load levels. In our study, we analyzed the relationship between viral load and outcome, introducing viral load as a continuous variable. Our results are in agreement with Wildeman *et al.*[10], Ruzek *et al.*[11] and Hjalmarsson A *et al*. [19]. Kamei S *et al*., who evaluated serial changes of intrathecal viral loads by chemiluminescence assay and nested PCR, did not find any evident association between viral load and outcome either [20]. Our results are also concordant with the study of Schloss *et al.,* comparing nested PCR to quantitative real-time PCR [21]. However, in that recently published study, not all patients were treated by acyclovir. In our cohort, all patients were treated by acyclovir and all samples were collected before introduction of antiviral treatment.

The absence of an association between HSE outcome and CSF HSV load could suggest that the pathogenicity of HSE may not be directly linked to extent of viral replication, but rather depends on the host response. This hypothesis is consistent with the fact that IgG levels were described to be significantly higher in patients with good prognostic than in patients with poor outcome [19].

Surprisingly, in our study, we found a significant association between a high red cell count in CSF and severe morbidity. To our knowledge, this association had not been described previously. It can be hypothesized that a higher red cell count in CSF could reflect more severe brain cell necrosis, which may be due to an intense and inappropriate immunological response of the host to viral aggression. Some experimental data suggested that an intense and inappropriate inflammatory response could be harmful, and might explain the higher risk of morbidity [22,23]. However, this hypothesis remains to be further elucidated.

Our study had several limitations. First, although our sample size was greater than in other studies [8,10,11], the number of patients with available CSF samples was still small. This limitation is due to the low prevalence of HSE. Thus, due to the lack of statistical power, we may not have identified a potentially existing association between HSV load and HSE outcome. However, we were able to identify factors that are usually reported to be associated with poor HSV outcome in the literature [1,2,5,6]. Second, the risk of selection bias occurrence in our study was low. Although not all patients with HSE were enrolled, those with and without available CSF samples did not statistically differ in their main characteristics. We observed a higher mortality rate in patients with available CSF samples than in those without. The reason for this difference in mortality is unclear. However, this did not bias our findings regarding the impact of HSV load on HSV outcome. Third, ideally, the impact of CSF HSV load on HSV encephalitis should be examined at the onset of first symptoms. This is, however, unfeasible and unrealistic. Finally, it can be speculated that retrospective determination of HSV load in frozen samples is not accurate and may lead to the absence of an association between viral load and HSE outcome. However, Revello *et al.* showed the absence of genetic matter loss in samples frozen at −20°C [9]. Jerome *et al.*, measured HSV loads in the same samples directly after sampling and after storage at 4°C for 16 months. HSV loads in freshly analyzed samples did not differ from those that had been stored at 4°C [24]. It is to note that in this last study, three positive specimens were negative upon retesting after storage. All these samples contained very low levels of virus even in the original quantitation and the authors estimated that the negative results may represent the inherent inaccuracy of quantitation of viral DNA in samples with low levels of virus rather than a true loss of viral DNA. In our study, we found 5 negative samples by quantitative PCR, although they were positive with qualitative PCR. We think that we encountered the same phenomenon as Jerome *et al.*: accurate quantitation of viral DNA is not possible in samples with low levels of virus. In addition, we sometimes had to work with low quantities of CSF due to limited availability of samples. This occurred more frequently in patients whose samples gave negative results by quantitative PCR. In one of these 5 patients, enough CSF was available to re-perform a real time qualitative PCR, which was positive at the 39^th^ cycle, corresponding to a low viral load. Thus, we can consider that these false negative results by quantitative PCR do not bias our results, because they effectively correspond to low HSV loads.

## Conclusions

In the present study, we found no relationship between poor outcome in patients suffering from HSV encephalitis and HSV load in CSF prior to acyclovir treatment initiation. But we found a relation between poor outcome and the presence of red blood cells in CSF. Our results suggest that, in herpetic meningo-encephalitis, severity is probably not due to the intensity of viral replication. Whether it is related to other physiopathological causes, such as intense and inappropriate inflammatory responses, remains to be elucidated. According to our results and that of similar studies, quantitative real-time PCR is not useful to diagnose HSE. Qualitative real-time PCR assay is perfectly suited.

## Abbreviations

HSE: *Herpes simplex* encephalitis; HSV: *Herpes simplex* virus; CSF: Cerebrospinal fluid.

## Competing interests

The authors declare no competing interests concerning this article.

## Authors’ contributions

JP collected the data, performed the quantitative PCR, the statistical analysis and wrote the manuscript; KC performed the statistical analysis, helped to the interpretation of the results and to the redaction of the manuscript; AD helped to identify the CSF samples, coordinated the realization of the quantitative PCR and helped to the redaction of the manuscript; HM gave a critical analysis of the work; HG gave a critical analysis of the work; ES gave a critical analysis of this work; YY had the idea of the study, coordinated its realization, verified the statistical analysis and wrote the manuscript. All authors read and approved the final manuscript.

## Pre-publication history

The pre-publication history for this paper can be accessed here:

http://www.biomedcentral.com/1471-2334/12/356/prepub

## References

[B1] Whitley RJ Viral encephalitis N Engl J Med 1990 323 4 242 250 10.1056/NEJM199007263230406 2195341

[B2] Whitley RJ Herpes simplex encephalitis: adolescents and adults Antiviral Res 2006 71 2–3 141 148 16675036 10.1016/j.antiviral.2006.04.002

[B3] Gordon B Selnes OA Hart J Jr Hanley DF Whitley RJ Long-term cognitive sequelae of acyclovir-treated herpes simplex encephalitis Arch Neurol 1990 47 6 646 647 10.1001/archneur.1990.00530060054017 2346392

[B4] Kapur N Barker S Burrows EH Ellison D Brice J Illis LS Scholey K Colbourn C Wilson B Loates M Herpes simplex encephalitis: long term magnetic resonance imaging and neuropsychological profile J Neurol Neurosurg Psychiatry 1994 57 11 1334 1342 10.1136/jnnp.57.11.1334 7964808 PMC1073183

[B5] Raschilas F Wolff M Delatour F Chaffaut C De Broucker T Chevret S Lebon P Canton P Rozenberg F Outcome of and prognostic factors for herpes simplex encephalitis in adult patients: results of a multicenter study Clin Infect Dis 2002 35 3 254 260 10.1086/341405 12115090

[B6] Whitley RJ Alford CA Hirsch MS Schooley RT Luby JP Aoki FY Hanley D Nahmias AJ Soong SJ Vidarabine versus acyclovir therapy in herpes simplex encephalitis N Engl J Med 1986 314 3 144 149 10.1056/NEJM198601163140303 3001520

[B7] Nahmias AJ Whitley RJ Visintine AN Takei Y Alford CA Jr Herpes simplex virus encephalitis: laboratory evaluations and their diagnostic significance J Infect Dis 1982 145 6 829 836 10.1093/infdis/145.6.829 6282983

[B8] Domingues RB Lakeman FD Mayo MS Whitley RJ Application of competitive PCR to cerebrospinal fluid samples from patients with herpes simplex encephalitis J Clin Microbiol 1998 36 8 2229 2234 9665997 10.1128/jcm.36.8.2229-2234.1998 PMC105021

[B9] Revello MG Baldanti F Sarasini A Zella D Zavattoni M Gerna G Quantitation of herpes simplex virus DNA in cerebrospinal fluid of patients with herpes simplex encephalitis by the polymerase chain reaction Clin Diagn Virol 1997 7 3 183 191 10.1016/S0928-0197(97)00269-9 9126688

[B10] Wildemann B Ehrhart K Storch-Hagenlocher B Meyding-Lamade U Steinvorth S Hacke W Haas J Quantitation of herpes simplex virus type 1 DNA in cells of cerebrospinal fluid of patients with herpes simplex virus encephalitis Neurology 1997 48 5 1341 1346 10.1212/WNL.48.5.1341 9153470

[B11] Ruzek D Piskunova N Zampachova E High variability in viral load in cerebrospinal fluid from patients with herpes simplex and varicella-zoster infections of the central nervous system Clin Microbiol Infect 2007 13 12 1217 1219 10.1111/j.1469-0691.2007.01831.x 17953699

[B12] Jennett B Bond M Assessment of outcome after severe brain damage Lancet 1975 1 7905 480 484 46957 10.1016/s0140-6736(75)92830-5

[B13] Teasdale G Jennett B Assessment of coma and impaired consciousness A practical scale. Lancet 1974 2 7872 81 84 10.1016/s0140-6736(74)91639-0 4136544

[B14] Knaus WA Zimmerman JE Wagner DP Draper EA Lawrence DE APACHE-acute physiology and chronic health evaluation: a physiologically based classification system Crit Care Med 1981 9 8 591 597 10.1097/00003246-198108000-00008 7261642

[B15] Mac Cabe W Jackson W Gram negative bacteriema: etiology and ecology Arch Intern Med 1962 110 83 91 10.1001/archinte.1962.03620190085013 14450740

[B16] Poissy J Wolff M Dewilde A Rozenberg F Raschilas F Blas M Georges H Chaffaut C Yazdanpanah Y Factors associated with delay to acyclovir administration in 184 patients with herpes simplex virus encephalitis Clin Microbiol Infect 2009 15 6 560 564 10.1111/j.1469-0691.2009.02735.x 19392906

[B17] Whitley R Arvin A Prober C Burchett S Corey L Powell D Plotkin S Starr S Alford C Connor J A controlled trial comparing vidarabine with acyclovir in neonatal herpes simplex virus infection. Infectious Diseases Collaborative Antiviral Study Group N Engl J Med 1991 324 7 444 449 10.1056/NEJM199102143240703 1988829

[B18] Whitley RJ Soong SJ Dolin R Galasso GJ Ch'ien LT Alford CA Adenine arabinoside therapy of biopsy-proved herpes simplex encephalitis. National Institute of Allergy and Infectious Diseases collaborative antiviral study N Engl J Med 1977 297 6 289 294 10.1056/NEJM197708112970601 195208

[B19] Hjalmarsson A Granath F Forsgren M Brytting M Blomqvist P Skoldenberg B Prognostic value of intrathecal antibody production and DNA viral load in cerebrospinal fluid of patients with herpes simplex encephalitis J Neurol 2009 256 8 1243 1251 10.1007/s00415-009-5106-6 19353228

[B20] Kamei S Takasu T Morishima T Mizutani T Serial changes of intrathecal viral loads evaluated by chemiluminescence assay and nested PCR with aciclovir treatment in herpes simplex virus encephalitis Intern Med 2004 43 9 796 801 10.2169/internalmedicine.43.796 15497513

[B21] Schloss L Falk KI Skoog E Brytting M Linde A Aurelius E Monitoring of herpes simplex virus DNA types 1 and 2 viral load in cerebrospinal fluid by real-time PCR in patients with herpes simplex encephalitis J Med Virol 2009 81 8 1432 1437 10.1002/jmv.21563 19551833

[B22] Kurt-Jones EA Belko J Yu C Newburger PE Wang J Chan M Knipe DM Finberg RW The role of toll-like receptors in herpes simplex infection in neonates J Infect Dis 2005 191 5 746 748 10.1086/427339 15688290

[B23] Kurt-Jones EA Chan M Zhou S Wang J Reed G Bronson R Arnold MM Knipe DM Finberg RW Herpes simplex virus 1 interaction with Toll-like receptor 2 contributes to lethal encephalitis Proc Natl Acad Sci U S A 2004 101 5 1315 1320 10.1073/pnas.0308057100 14739339 PMC337050

[B24] Jerome KR Huang ML Wald A Selke S Corey L Quantitative stability of DNA after extended storage of clinical specimens as determined by real-time PCR J Clin Microbiol 2002 40 7 2609 2611 10.1128/JCM.40.7.2609-2611.2002 12089286 PMC120597

